# Recent incidence of type 1 diabetes mellitus in children 0–14 years in Newfoundland and Labrador, Canada climbs to over 45/100,000: a retrospective time trend study

**DOI:** 10.1186/1756-0500-5-628

**Published:** 2012-11-12

**Authors:** Leigh A Newhook, Sharon Penney, Jackie Fiander, Jeff Dowden

**Affiliations:** 1 Janeway Pediatric Research Unit, Memorial University, 300 Prince Philip Drive, St. John’s, NL, A1B 3V6, Canada; 2 Janeway Children’s Health and Rehabilitation Centre, Eastern Health, St. John’s, NL, Canada; 3 Newfoundland and Labrador Centre for Health Information, St. John’s, NL, Canada

**Keywords:** Type 1 diabetes mellitus, Incidence, Newfoundland and Labrador, Canada, Epidemiology

## Abstract

**Background:**

To study and update the provincial incidence of type 1 diabetes mellitus (T1DM) in Newfoundland and Labrador (NL), a province of Canada with a very high incidence previously reported in 2006, and one of the highest incidences reported worldwide. This is a retrospective time trend study of the incidence of T1DM, in children aged 0–14 years from 1987–2010 inclusive.

**Findings:**

Over the study period 931 children aged 0–14 years were diagnosed with T1DM. The incidence of T1DM in this population over the period 1987 – 2010 inclusive was 37.7 per 100,000 per year (95% CI 35. 3, 40.2)

The incidence from 2007–2010 was 49.9 per 100,000 per year (95% CI 42.2, 57.6). The incidence over this 24 year period increased by a factor of 1.03 per 100,000 per year.

**Conclusion:**

NL has one of the highest incidences of T1DM reported worldwide. Potential reasons for the very high incidence could be related to the unique genetic background of the population, northern latitude and vitamin D insufficiency, low breastfeeding rates, and high rates of cesarean section.

## Findings

### Background

In 2006, we reported a “very high” incidence of 35.1 per 100,000 per year of T1DM in the 0–14 year age group over the period 1987–2005 in Newfoundland and Labrador (NL) [1]. Other findings showed a significant difference between the incidence for boys versus girls in the 0-4-year age group (31.61 and 19.05 respectively per 100,000 per year, p=0.0013) and the estimated rate of incidence increase was 0.783 per 100,000 children ages 0–14 years per year [1]. The rates of T1DM in NL appear to be continually rising and this update was done to investigate this hypothesis.

### Methods

This retrospective time trend study was performed across the Canadian province of NL. All children with T1DM who live in NL and were diagnosed with T1DM from 1987 to 2010 are included in the study. Patient sex, month of onset of diabetes, month of birth, and age of onset were collected and recorded by diabetes nurse educators in each facility that cares for children with T1DM and reports of new cases are faxed to the research nurse monthly at the Janeway Pediatric Research Unit at the tertiary care Janeway Children’s Health and Rehabilitation Centre, Eastern Health, in St. John’s. Classification, case definition and ascertainment are described elsewhere [1,2].

#### Ethical considerations

The Memorial University of Newfoundland and Labrador Human Investigations Committee and each of the hospital board’s ethics committees approved this study.

#### Statistical analysis

The data described is incidence per 100,000 of the age-specific population obtained from Canadian census data [3]. Age-standardized rates were computed across specified time periods using 1991 Canadian Census population as standard. 95% confidence intervals were derived using Poisson distribution. Poisson log-linear regression was used to examine trends in annual incidence rates.

### Results

A total of 931 cases were identified (Table 1); there were 485 males and 446 females with 1.1:1 ratio (M:F). Two cases did not specify gender. Incidence was significantly higher for males than females in the 0–4 year age group (m=32.7 vs. f=21.7 per 100,000 per year; p=0.006). Yearly incidence rates for ages 0–4 increased by a factor of 1.03 per 100,000 (95% CI: 1.01-1.04); whereas rates for ages 5–9 and 10–14 increased by factors of 1.04 per 100,000 (1.02-1.05), and 1.02 per 100,000 (1.01-1.04), respectively.

**Table 1:** Incidence, gender, and time trends of type 1 diabetes mellitus (0–14 yr) per 100,000 individuals in Newfoundland and Labrador, Canada, 1987-2010

**Years**	**Sex**	**0-14 yr (n)**	**95% CI**	**0-4 yr (n)**	**95% CI**	**5-9 yr (n)**	**95% CI**	**10-14 yr (n)**	**95% CI**
**1987-2010**	M+F	37.7 (931)	35.3-40.2	27.3 (196)	23.6-31.4	42.8 (350)	38.5-47.6	41.1 (385)	37.1-45.4
**1987-2010**	M	38.3 (485)	35.0-41.9	32.7* (120)	**27.1-39.1**	40.0 (167)	34.2-46.5	41.2 (198)	35.7-47.3
**1987-2010**	F	37.0 (446)	33.6-40.6	21.7* (76)	**17.1-27.1**	45.8 (183)	39.4-53.0	41.0 (187)	35.3-47.3
**1987-1991**	M+F	29.9^†^ (207)	24.4-35.5	21.3 (42)	15.3-28.7	33.2 (75)	26.1-41.7	35.3 (90)	28.4-43.4
**1992-1996**	M+F	31.9^†^ (190)	26.1-37.8	27.3 (47)	20.1-36.3	37.3 (73)	29.2-46.9	31.2 (70)	24.3-39.4
**1997-2001**	M+F	40.9^†^ (203)	34.3-43.4	28.0 (38)	19.8-38.4	46.7 (75)	36.7-58.5	48.1 (90)	38.7-59.1
**2002-2006**	M+F	42.3^†^ (178)	35.5-49.2	30.2 (36)	21.2-41.8	50.5 (68)	39.2-64.0	46.4 (74)	36.4-58.2
**2007-2010**	M+F	49.9^†^ (153)	42.2-57.6	35.8 (33)	24.6-50.2	59.1 (59)	45.0-76.3	54.7 (61)	41.9-70.4

CI, confidence interval; n, specified size of age group; F, Female; M, Male; ^†^ rates age-standardized to 1991 Canadian Census; * p=0.006.

Trends were not significantly different across age groups. Age-standardized rates for census divisions ranged from 18.8 per 100,000 per year on the South Coast to 45.8 per 100,000 per year in Central NL (Table 2).

**Table 2:** **Incidence of type 1 diabetes mellitus (0–14 yr) by geographical census division**^**±**^**, Newfoundland and Labrador, Canada, 1987-2010**

**Census division**	**Incidence rate**	**95% CI**
(1) Avalon Peninsula	40.2	34.9-45.4
(2) Burin Peninsula	33.3	24.6-42.0
(3) South Coast	18.8	10.7-26.9
(4) St. George’s	33.7	24.7-42.6
(5) Humber District	33.6	25.7-41.5
(6) Central	45.8	37.1-54.5
(7) Bonavista/Trinity	33.3	25.3-41.4
(8) Notre Dame Bay	37.8	29.9-45.7
(9) Northern Peninsula	40.6	30.9-50.3
(10) Labrador	39.9	31.1-48.8

^±^ Rates age-standardized to 1991 Canadian Census.

### Discussion

This study represents an updated analysis on the population of children 0–14 years with T1DM from the province of NL, Canada, confirming a very high and increasing incidence over the 24-year study period. The incidence from 1987 to 2010 inclusive is 37.7 per 100,000 per year, one of the highest reported worldwide. The incidence from 2007–2010 was 49.9 per 100,000 per year. The incidence increased significantly in all age groups and there are significantly more males diagnosed with T1DM than females in the 0–4 year age group. A male excess is more often found in populations with a high incidence of T1DM [4,5].

The incidence of T1DM is increasing worldwide, especially for the youngest ages and those with moderate genetic susceptibility. The rapidly rising incidence cannot be explained by genetic susceptibility alone and is hypothesized to be due to environmental, lifestyle, and or epigenetic factors that are triggering or accelerating the onset and progression of the disease [6]. The increasing incidence that we are reporting for NL is similar to reports from other large epidemiologic studies including DiaMOND [7], EURODIAB [8], and SEARCH [9]. Other regions of Canada have also reported high rates of T1DM but data on trends over time are not available [10,11]. Although we do not have an explanation for the unusually high incidence of T1DM in NL, there are several factors that have been identified in other regions that may be important for this population. Genetic factors are likely to be important and research is underway to explore this further. Newfoundland is an island and Labrador, although part of mainland Canada, is relatively isolated. The population of NL has a well-documented immigration history with 96% of residents having ancestors either from southeast Ireland or southwest England [12], as well as low intraprovincial migration rates [12,13] and founder effects [14,15].

The rising incidence over a relatively short period of time however suggests environmental factors are contributing to the increase in a genetically at risk population. Potential environmental risk factors that have been identified in other populations which may be associated with T1DM in NL include early infant nutrition, perinatal factors, northern latitude, and vitamin D deficiency. NL has the lowest rates of initiation and duration of breastfeeding in Canada [16]; exclusive breastfeeding has been shown have a weak protective association with T1DM risk [17-19]. Vitamin D deficiency has been associated with increased risk of T1DM [20,21], vitamin D supplementation may be protective against T1DM [22] and UVB irradiation and northern latitude are also associated factors [23]. NL has a northern latitude (49°N) and erythemal UVB radiation may be geospatially associated with the incidence of T1DM NL [24]. Vitamin D insufficiency has been documented in the population [25]. NL has a high rate of birth by C-section as compared to other regions in Canada. The provincial rate of births by C-section was 30.9% in 2005–2006 versus the Canadian rate of 26.3% [26]. The rates of C-section have increased in NL to 33% in 2010 [27]. Recent results from a population-based case–control study showed that birth by cesarean section is associated with T1DM in NL(HR 1.41, p=0.015) [28]. A recent meta-analysis of 20 studies found that the combined effect of C-section delivery on T1DM risk was 1.23 (95% CI 1.15-1.32) [29].

### Conclusions

The results from this research continue to show a very high and increasing incidence. We continue to research the genetics, epidemiology and environmental risk factors of the disease in this population with hopes of contributing to a better understanding of the etiology and pathogenesis of T1DM. Research into the relationship and underlying reasons for the association between birth by c-section and T1DM risk are continuing, as well as research on vitamin D insufficiency, early infant nutrition and growth. NL is a unique and important place to study T1DM with its geography, stable population, genetic founder effects and unusually high incidence.

## Competing interests

There are no conflicts of interest to disclose.

## Authors’ contributions

LN is the principle investigator and was responsible for the intellectual conception and design of the study, funding application, manuscript preparation and is the guarantor of the research. SP was the research nurse who was responsible for study approvals, coordination of the study as well as gathering, confirming and entering of data. JF was responsible for overall coordination of the study and contributed intellectually to the methods, database development, logistics, approvals, and funding application submission. JD was responsible for the statistical analysis. All authors contributed to and approved the final manuscript submission.
